# Ganciclovir Pharmacokinetics and Individualized Dosing Based on Covariate in Lung Transplant Recipients

**DOI:** 10.3390/pharmaceutics14020408

**Published:** 2022-02-13

**Authors:** Eliška Dvořáčková, Martin Šíma, Jakub Petrus, Eva Klapková, Petr Hubáček, Jiří Pozniak, Jan Havlín, Robert Lischke, Ondřej Slanař

**Affiliations:** 1 Department of Pharmacology, First Faculty of Medicine, Charles University and General University Hospital in Prague, 128 00 Prague, Czech Republic; eliskadvorackova@seznam.cz (E.D.); ondrej.slanar@lf1.cuni.cz (O.S.); 2 Department of Medical Chemistry and Clinical Biochemistry, Second Faculty of Medicine, Charles University in Prague and Motol University Hospital, 150 06 Prague, Czech Republic; jakubpet@gmail.com (J.P.); eva.klapkova@fnmotol.cz (E.K.); 3 Department of Medical Microbiology, Second Faculty of Medicine, Charles University in Prague and Motol University Hospital, 150 06 Prague, Czech Republic; petr.hubacek@fnmotol.cz; 4 Prague Lung Transplant Program, 3rd Department of Surgery, First Faculty of Medicine, Charles University in Prague and Motol University Hospital, 150 06 Prague, Czech Republic; jiri.pozniak@fnmotol.cz (J.P.); jan.havlin@fnmotol.cz (J.H.); robert.lischke@fnmotol.cz (R.L.)

**Keywords:** ganciclovir, lung transplant recipients, cystic fibrosis, therapeutic drug monitoring, covariates

## Abstract

The aim of this prospective study was to evaluate the pharmacokinetics of ganciclovir in lung transplant recipients, to explore its covariates, and to propose an individualized dosing regimen. Ganciclovir was administered according to the protocol in a standardized intravenous dose of 5 mg/kg twice daily. Serum ganciclovir concentrations were monitored as a trough and at 3 and 5 h after dosing. Individual ganciclovir pharmacokinetic parameters were calculated in a two-compartmental pharmacokinetic model, while regression models were used to explore the covariates. Optimal loading and maintenance doses were calculated for each patient. In lung transplant recipients (n = 40), the median (IQR) ganciclovir total volume of distribution and clearance values were 0.65 (0.52–0.73) L/kg and 0.088 (0.059–0.118) L/h/kg, respectively. We observed medium-to-high inter-individual but negligible intra-individual variability in ganciclovir pharmacokinetics. The volume of distribution of ganciclovir was best predicted by height, while clearance was predicted by glomerular filtration rate. Bodyweight-normalized clearance was significantly higher in patients with cystic fibrosis, while distribution half-life was reduced in this subgroup. On the basis of the observed relationships, practical nomograms for individualized ganciclovir dosing were proposed. The dosing of ganciclovir in patients with cystic fibrosis requires special caution, as their daily maintenance dose should be increased by approximately 50%.

## 1. Introduction

Ganciclovir is an antiviral agent with broad activity against herpes viruses, including cytomegalovirus. It is indicated for the prophylaxis and treatment of herpesvirus infection in immunocompromised patients, including lung transplant recipients, in whom cytomegalovirus infection is associated with premature graft failure and decreased overall survival [1,2]. In routine clinical practice, ganciclovir dosing is adjusted according to the patient’s weight, renal function and indication (prophylaxis or treatment) [3]. However, this approach may vary among different institutions, depending on the dosing algorithm locally adopted. Several institutions extrapolate the results generated in studies with patients with AIDS or renal transplant recipients [4]. The method used for renal function estimation is another potential source of variability among clinical centers. Therapeutic drug monitoring (TDM) may be helpful for dose adjustment to maintain efficacious drug levels related to viral inhibitory concentrations, although the timely availability of appropriate bioanalytical assays of antiviral drugs is limited [5]. Although there is currently little consensus on the therapeutic range that would optimally predict clinical outcomes and toxicity, subtherapeutic levels of ganciclovir may lead to the selection of resistant strains (e.g., with mutations in the UL97 gene–viral thymidine kinase) with subsequent treatment failure [6]. By contrast, high exposure to ganciclovir increases the risk of myelosuppression and neurotoxicity [7,8].

Cystic fibrosis is one of the major indications for lung transplantation [9]. Although changes in the drug disposition can be expected in these patients, no pharmacokinetic study on ganciclovir pharmacokinetics has been published apart, from observational data for C_max_, C_min_ and AUC from 12 patients with cystic fibrosis [10]. Ganciclovir TDM may be especially beneficial for patients with highly variable pharmacokinetic profiles, such as patients with unstable renal function or cystic fibrosis.

Ganciclovir is a cyclic analogue of endogenous purine nucleoside guanosine, with an intracellular half-life of 16.5 h [11]. The drug has low protein binding (1–2%), a rapid distribution phase (0.23 h) and a terminal serum half-life of 2–4 h. Renal clearance is the dominant form of elimination. Most of the drug is eliminated via glomerular filtration, with more than 80% of the administered dose found in the urine unchanged [12].

Although ganciclovir is routinely used for prophylaxis and treatment in lung recipients, its individual pharmacokinetic parameters and covariates (sex, age, bodyweight, height, serum creatinine, serum cystatin C, liver enzymes, white blood cells, platelet count, concomitant pharmacotherapy and cystic fibrosis) have not yet been clearly addressed. Therefore, the objective of this prospective study was to evaluate the pharmacokinetics of ganciclovir in lung transplant recipients and to explore its covariates in order to propose an individualized ganciclovir dosing regimen prior to TDM.

## 2. Materials and Methods

### 2.1. Study Design

This was a prospective open-label (laboratory-blinded) pharmacokinetic study on adult patients treated with intravenous ganciclovir (Cymevene^®^; CHEPLAPHARM Arzneimittel GmbH, Greifswald, Germany) at the Third Department of Surgery, First Faculty of Medicine, Charles University in Prague and Motol University Hospital between January 2020 and July 2021. Patients were included if they met all the following inclusion criteria: age ≥18 years, had undergone lung transplantation and received antiviral prophylaxis with intravenous ganciclovir twice daily at least 48 h after transplantation. Patients treated with ganciclovir before lung transplantation, patients with combined transplantation, re-transplantation, and patients aged under 18 years were excluded from this study. All patients received basiliximab or antithymocyte globulin as induction immunosuppressive therapy. All patients also received triple drug immunosuppression with tacrolimus (Prograf^®^; Astellas Pharma s.r.o., Praha, Czech Republic), mycophenolate mofetil (CellCept^®^; Roche Registration GmbH, Grenzach-Wyhlen, Germany) and prednisone (Prednison^®^; Zentiva; Prague, Czech Republic)/methylprednisone (Solu-Medrol^®^; Pfizer, spol. s r.o., Prague, Czech Republic). Antiviral therapy for all patients was administered according to the standardized protocol. Steady-state whole-blood concentrations of ganciclovir were measured over a median of 9 days (2–28) after transplantation. The study was approved by the local Ethics Committee under No. EK- 11/20 and was conducted in accordance with the Declaration of Helsinki. Written informed consent was obtained from all subjects before any study-related procedures. Ganciclovir was initially administered at a dose of 5 mg/kg/12 h given through 60 min intravenous infusion at concentrations not exceeding 10 mg/mL. Whole-blood concentrations for pharmacokinetic analysis were taken as a trough (C_trough_) and at 3 (C_3_) and 5 h (C_5_) after the infusion was completed. Patients from whom a complete concentration-time profile was not collected were excluded from the study. Whole-blood samples (5 mL) were collected into serum collecting tubes without clot activators and immediately placed in the cold. The samples were then centrifuged at 4500× *g* for 10 min at 4 °C and serum aliquots were stored at −80 °C until analysis.

The following demographic, laboratory and clinical characteristics of the patients were recorded as potential covariates of ganciclovir pharmacokinetics: sex, age, body weight, height, serum creatinine, serum cystatin C (available only in some patients), alanine aminotransferase (ALT), gamma-glutamyl transferase (GGT), white blood cell and platelet counts, diagnosis of cystic fibrosis and co-medication with immunosuppressants (tacrolimus, mycophenolate mofetil, corticosteroids) or antimycotics (voriconazole-Vfend^®^; Pfizer Europe MA EEIG, Bruxelles, Belgien, fluconazole-Fluconazole^®^, Aurovitas, spol. s r.o., Prague, Czech Republic).

For each patient, the body mass index (BMI), body surface area (BSA) according to the DuBois formula, estimated glomerular filtration rate (eGFR) according to CKD-EPI creatinine and optionally according to CKD-EPI cystatin C equations were calculated [13,14,15].

### 2.2. Bioanalytical Assay

Liquid chromatography-mass spectrometry (LC-MS)-grade acetonitril, ammonium acetate, trifluoroacetic acid, acetic acid and trichloroacetic acid (HPLC grade) were obtained from Supelco and Honeywell (HPST, Prague, Czech Republic). Ganciclovir (reference standard) and deuterium-labelled internal standard ganciclovir-d5 were obtained from Toronto Research Chemicals Inc. (Toronto, ON, Canada). We used an Agilent Technologies 1290 Infinity II LC system, including an autosampler, binary pumps, and a thermostatted column compartment with 6470 Triple Quad (Agilent Technologies, Santa Clara, CA, USA). Other necessary equipment were MS 40+ Vacuum Products (Agilent Technologies, Santa Clara, CA, USA) and a nitrogen generator NM32LA (Peak Scientific, Inchinnan, UK). Sample separation was carried out on a reverse phase column Eclipse Plus C18, 1.8 μm, 3.0 × 50 mm (Agilent Technologies, Santa Clara, CA, USA). The column was operated at 35 °C. A chromatographic separation was achieved under gradient flow of eluents, initially in 95/5 mix of mobile phase (A) water and aqueous buffer (95/5, *v*/*v*) and (B) acetonitril and aqueous buffer (95/5, *v*/*v*). The set-up of the gradient is shown in Table 1. The autosampler was cooled at 7 °C. An aqueous buffer was prepared at this concentration (ammonium acetate 5 g/L, 2 mL/L trifluoroacetic acid, and 35 mL/L acetic acid). The total run time per sample was 4 min. For measurement, we used 10 µL of serum sample (control, calibrator) and 500 µL of internal standard (ganciclovir-d5 5 µg/L dissolved in 5% trichloroacetic acid). The sample was briefly mixed and then centrifuged 10 min at 3727 *g*. A total of 50 µL of the upper layer was then mixed with 950 µL of 5% trichloroacetic acid. A total of 5µL of prepared sample was injected into the column. The Agilent Jet Stream with electrospray ionization ion source operated in positive ion mode. The scan type used dynamic multiple reaction monitoring. The measurements used were: gas temperature at 250 °C, gas flow 8 L/min, nebulizer at 45 psi, sheath gas temperature 350 °C and sheath gas flow 11 L/min. The mass ion transitions for ganciclovir were 256.1 *m*/*z* → 152 *m*/*z* and for ganciclovir-d5 were 261.1 *m*/*z* → 152 *m/z*. Collision energies were 12 V for both analytes. Full validation according to the U.S. Food and Drug Administration (FDA) requirements was conducted [16]. The calibration curve was constructed by plotting the peak ratios of ganciclovir standard to the internal standard against the concentration of ganciclovir. The assay was linear (r^2^ was 0.9938) across the whole range of concentrations (0.1; 0.5; 1; 2.5; 5; 10; 20 mg/L). The intra- and inter-day accuracy and precision were evaluated in three QC samples (0.5; 2.5; 10 mg/L) by multiple analysis (n = 10). The intra-day and inter-day accuracy ranged from 0.86% to 1.16% and from 3.58% to 8.32%, respectively. The ranges of intra-day and inter-day precision were 6.21% to 8.90 and 1.39% to 1.50%, respectively. The limit of quantification was 0.1 mg/L. The intra- and inter-day accuracy was expressed as the relative error in % for LLOQ (n = 10) was 2.39% and 4.54%, respectively. The intra-day and inter-day precision for LLOQ was 7.04% and 14.02%, respectively. Detailed concentrations measured in the samples at each spiking level in the intra-day and inter-day accuracy and precision study are summarized in Table 2. The sample stability of ganciclovir was documented at room temperature or −20 °C for 7 days or 3 months, respectively. Sample stability after three freeze-thaw cycles was also evaluated. Maximal change of concentration was within ±5% during all stability tests.

### 2.3. Pharmacokinetic Analysis

Individual pharmacokinetic parameters of ganciclovir, namely central compartment volume of distribution (Vd_c_), total volume of distribution (Vd), clearance (CL), distribution half-life (t_1/2_α), elimination half-life (t_1/2_β), and 24 h area under the concentration-time curve (AUC_24_) were calculated in a two-compartmental pharmacokinetic model with first-order elimination kinetics based on individual demographic and clinical data and observed ganciclovir serum levels using MWPharm^++^ software (MediWare, Prague, Czech Republic). The ganciclovir pharmacokinetic data derived from Sommadossi et al. was used for a priori simulation of concentration-time profile in each patient [17]. These simulated pharmacokinetic profile curves were a posteriori individualized to maximize fitting with observed concentration points of each patient. The fitting was performed using the Bayesian method. The Bayesian approach defines all unknown parameters as random variables and via a large number of subsequent iterations, the variables are adapted, taking into account the physiological and substance properties to achieve maximal fitting of the simulated pharmacokinetic profile curve with the real measured concentration points in each patient. The goodness-of-fit was expressed using weighted sum of squares and root mean square values.

For patients whose set of ganciclovir levels (C_trough_, C_3_ and C_5_) was measured repeatedly during hospitalization, the pharmacokinetic parameters of ganciclovir were calculated separately from each set of concentrations. Only the PK parameters from the first drug concentration triplet (C_trough_, C_3_ and C_5_) were used for the analysis of covariates, while subsequent data sets were used for the analysis of intraindividual variability.

### 2.4. Statistical Analysis

Descriptive parameters of mean, standard deviation, coefficient of variation, median and interquartile range (IQR) were calculated using MS Excel 2010 (Microsoft Corporation, Redmond, DC, USA). The 95% confidence intervals (CI) for medians were calculated using the Bonett and Price method [18]. Mann–Whitney U-test or linear regression model were used to evaluate the relationships of individual ganciclovir pharmacokinetic parameters and categorical or continuous variables, respectively. Pharmacokinetic parameters obtained from one patient repeatedly during hospitalization were compared using Wilcoxon signed-rank test. Possible impact of immunosuppressants (taken by all patients at different doses) on ganciclovir pharmacokinetic parameters was evaluated in a dose-dependent manner using linear regression, while the effect of antimycotics (taken only by some patients) was evaluated dose-independently using the Mann–Whitney U-test, as described previously [19]. GraphPad Prism 8.2.1 software (GraphPad Inc., La Jolla, CA, USA) was used for all comparisons and *p*-levels < 0.05 were considered statistically significant.

### 2.5. Loading and Maintenance Dose Calculation

Optimal loading doses (LD) were calculated for each patient based on individual ganciclovir Vd values using the following formula: LD (mg) = ganciclovir Vd (L) × 7.75 mg/L. The maximum concentration of 7.75 mg/L was set as a midpoint of the proposed therapeutic range for peak ganciclovir levels (3–12.5 mg/L) [20].

Optimal daily maintenance doses (MD) were calculated for each patient based on individual ganciclovir CL values using the following formula: MD (mg/day) = 24 h × ganciclovir CL (L/h) × 6.75 mg/L. The steady-state concentration of 6.75 mg/L was set as a midpoint of the proposed therapeutic range for ganciclovir both at trough and peak levels (1–12.5 mg/L) [20].

## 3. Results

There were 54 patients enrolled in the study. Fourteen patients were excluded due to discontinuation of ganciclovir therapy, deviations in sampling times, or missing samples. Therefore, 40 patients were included in the pharmacokinetic analysis. The demographic, laboratory and clinical characteristics of the patients are summarized in Table 3. Among the patients included in the pharmacokinetic analysis, only one subject received CVVHD support, while none of the patients needed support with extracorporeal membrane oxygenation. The ganciclovir dose ranged from 100 mg/day to 1000 mg/day. All the patients were concomitantly treated with immunosuppressive drugs (tacrolimus, mycophenolate mofetil and prednisone or methylprednisolone). The median (IQR) doses of tacrolimus, mycophenolate mofetil and corticoid (expressed as prednisone equivalent dose) were 6 (0–14) mg, 1500 (250–3000) mg and 30 (20–63) mg, respectively. Four patients were treated with voriconazole (400 mg/day) and three patients received fluconazole (400 mg/day). There were five patients with cystic fibrosis in our study group.

**Figure 1:**
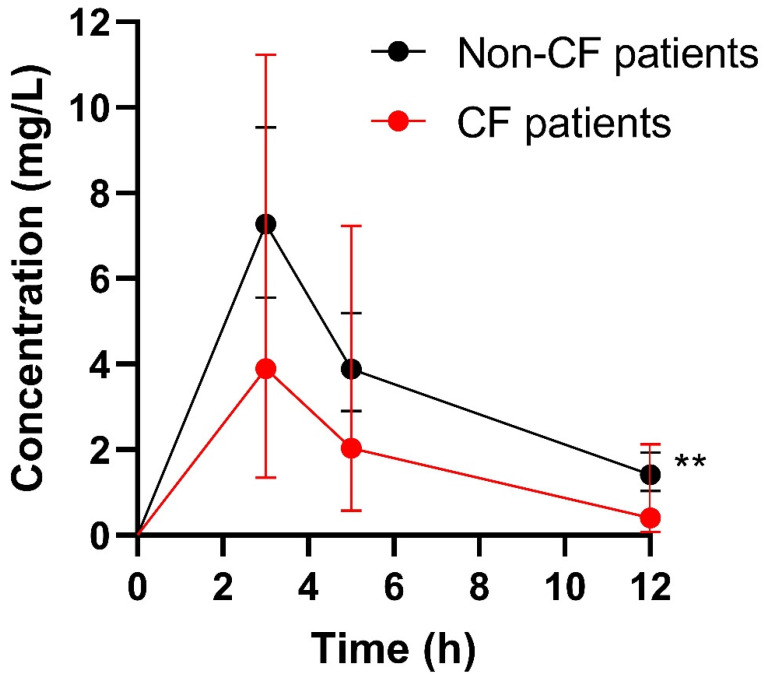
Ganciclovir pharmacokinetic profiles in both cystic fibrosis (CF) and non-cystic fibrosis (non-CF) patients. Data are expressed as geomeans (95% CI). ** Significantly different (*p* = 0.0085).

In total, 132 ganciclovir serum concentrations were included in the analysis. In four patients, the ganciclovir concentration set (C_trough_, C_3_ and C_5_) was measured twice during hospitalization. The ganciclovir pharmacokinetic profiles of both cystic fibrosis and non-cystic fibrosis patients are shown in Figure 1. The individual pharmacokinetic parameters of ganciclovir used in the study are summarized in Table 4. The median (IQR) weighted sum of squares and root mean square values were 4. 23 (1.19–14.04) and 0.97 (0.90–0.99), respectively. We observed medium-to-high inter-individual variability of pharmacokinetic parameters normalized per kg of body weight, as demonstrated by coefficients of variation of 19%, 59%, 52%, 68% and 79% for Vd_c_, Vd, CL, t_1/2_α and t_1/2_β, respectively. By contrast, there were no significant differences in ganciclovir pharmacokinetic parameters obtained from the same patients (n = 4) repeatedly during hospitalization (*p*-value of 0.5000, >0.9999, >0.9999, 0.5000 and 0.8750 for Vd_c_, Vd, CL, t_1/2_α and t_1/2_β, respectively), which indicates negligible intra-individual variability.

All the sampling time points (C_3_, C_5_ and C_trough_) were significantly associated with ganciclovir total exposure (AUC); however, AUC was best predicted by the peak level (r^2^ was 0.7720, 0.3184 and 0.2580 for C_3_, C_5_ and C_trough_, respectively).

**Figure 2:**
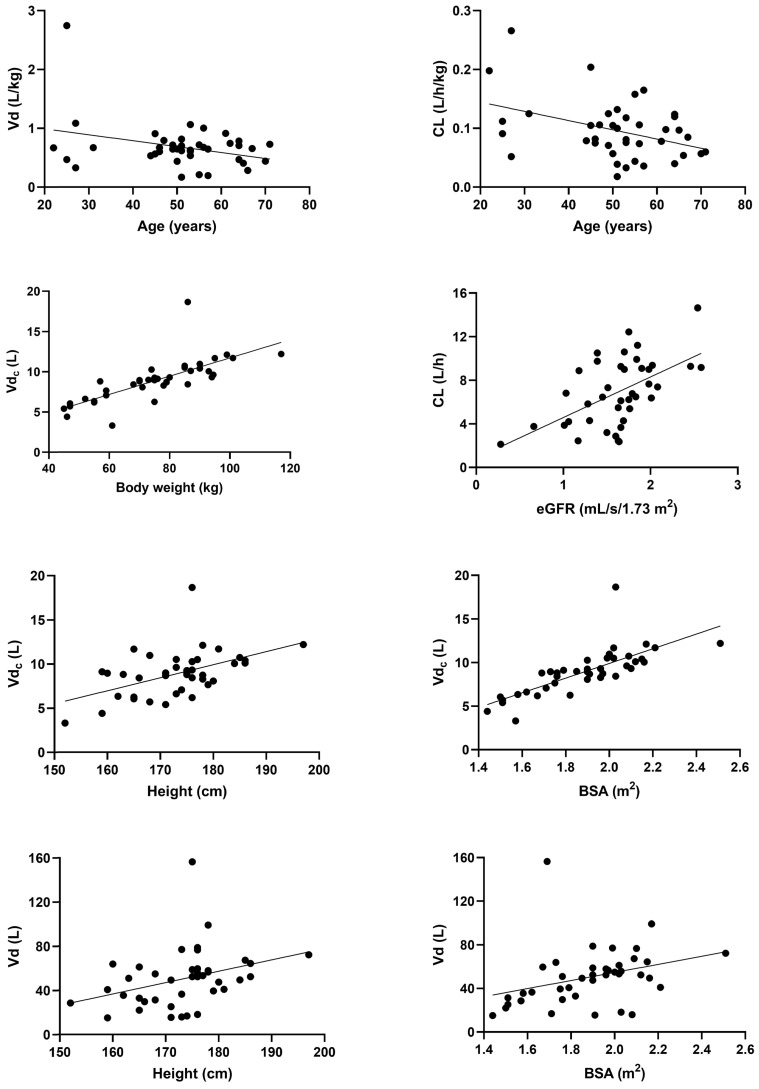
Relationships between ganciclovir pharmacokinetic parameters and its main covariates. Vd_c_—central volume of distribution, Vd—total volume of distribution, CL—clearance, BSA—body surface area, eGFR—estimated glomerular filtration rate according to the CKD-EPI creatinine equation.

Both Vd and CL normalized by body weight were significantly and negatively related to age (*p* = 0.0439 and *p* = 0.0116, respectively). Males showed significantly higher bodyweight-normalized Vd than females (median value 0.69 vs. 0.55 L/kg; *p* = 0.0330). Vd_c_ was significantly related to bodyweight, height, BSA and BMI (*p* < 0.0001, *p* = 0.0011, *p* < 0.0001, and *p* < 0.0001, respectively), while total Vd increased significantly only with height (*p* = 0.0297) and BSA (*p* = 0.0386). CL was significantly related only to eGFR (*p* < 0.0001). For the patients whose cystatin C level was measured (n = 24), the predictive performance of creatinine- and cystatine C-based CKD-EPI formulas for the estimation of glomerular filtration rate was compared. In this sense, creatinine-based estimates performed slightly better numerically (*p* = 0.0010, r^2^ = 0.3952 vs. *p* = 0.0033, r^2^ = 0.3309). Both ALT and GGT were not significantly related to the pharmacokinetics of ganciclovir. Ganciclovir exposure (AUC_24_) was also not associated with either white blood cell or platelet counts. Bodyweight-normalized CL was significantly higher in patients with cystic fibrosis, while distribution half-life was reduced in patients with this diagnosis (see Table 4). There was also a trend towards increased volume of distribution in the cystic fibrosis patients. We observed no dose-dependent drug interaction between immunosuppressive therapy and ganciclovir weight-normalized pharmacokinetic parameters. The dose-independent analysis also did not show any impact of antimycotic therapy on ganciclovir disposition. The main observed relationships between ganciclovir’s pharmacokinetic parameters and its covariates are showed in Figure 2.

**Figure 3:**
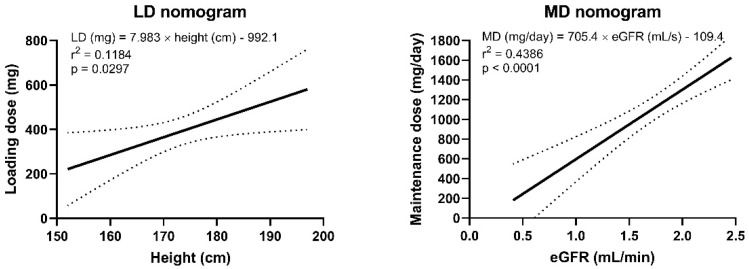
Nomograms for the calculation of the loading dose (LD) and the daily maintenance dose (MD) of ganciclovir according to height and estimated glomerular filtration rate according to the creatinine CKD-EPI formula, respectively. The dashed lines represent the 95% confidence interval.

Based on the regression analysis, height was shown to be the most predictive parameter for ganciclovir Vd and, consequently, for LD. Thus, CL and MD were best predicted by eGFR according to the creatinine CKD-EPI equation. Based on these relations, the optimal estimated LD was defined according to the following equation: LD (mg) = 7.988 × height (cm)–992.1. Optimal estimated daily MD was described as: MD (mg/day) = 705.4 × eGFR (mL/s)–109.4. These relationships were used to construct the dosing nomograms for more convenient clinical use (Figure 3). The median (95% CI) LD ganciclovir and daily MD were 2.31 (2.24–2.39) mg per cm of height and 643.88 (638.18–649.59) mg per 1 mL/s of eGFR, respectively. Subsequently, we simulated the administration of the dose recommended by the nomograms in model subjects with the pharmacokinetic data of each individual enrolled in the study. After the simulated LD administration, 32 (80%) of the patients reached the target range for ganciclovir peak concentrations (3–12.5 mg/L), 7 (17.5%) were above and 1 (2.5%) was below the range, while after the simulated administration of MD, 33 (82.5%) of the patients reached the target range for ganciclovir levels in the whole interval (1–12.5 mg/L), while 17 (17.5%) were above and none (0%) were below the range.

## 4. Discussion

Cytomegalovirus is a leading cause of infection in lung transplant recipients and it is associated with significant morbidity and mortality [21]. Adequate cytomegalovirus dosing is therefore of great importance, as low serum concentrations of ganciclovir should be avoided to minimize the risk of resistance development [22]. To ensure adequate exposure to ganciclovir, TDM could be applied to optimize ganciclovir serum concentrations during treatment or prophylaxis. Although the target ganciclovir levels that should be achieved during therapy have not yet been unequivocally defined [23], the therapeutic ranges frequently used for peak and trough ganciclovir concentrations in clinical practice are 3–12 and 1–3 mg/L, respectively [20]. The monitoring of ganciclovir exposure especially, in high-risk patient groups with unpredictable pharmacokinetics, i.e., patients with unstable renal function, solid organ transplant recipients, or patients not responding to treatment as expected, has been suggested [24].

In this study, we reviewed the pharmacokinetics of ganciclovir in patients after lung transplantation, on whom TDM was performed. In total, 132 samples were received from 40 lung transplant recipients. This represents one of the largest data sets describing ganciclovir pharmacokinetics in this vulnerable population.

We observed medium-to-high inter-individual variability of pharmacokinetic parameters, which was similar to the results of Märtson et al. and Galar et al. [24,25]. By contrast, there were no significant differences in the ganciclovir pharmacokinetic parameters obtained from the same patients.

We observed an increase in ganciclovir CL of approximately 50% in patients with cystic fibrosis. Although the patients with cystic fibrosis were significantly younger than the patients not suffering from this disease and we found a negative relationship between age and bodyweight-normalized ganciclovir CL in this subgroup, we assume that the independent covariate of ganciclovir CL is cystic fibrosis, because there was no relationship between age and ganciclovir CL in the patients not suffering from cystic fibrosis. Although the pharmacokinetics of ganciclovir have not been described previously in patients with cystic fibrosis, our findings correspond well with the theoretical assumption that ganciclovir enhances the clearance of renally eliminated compounds. It was previously shown that cystic fibrosis leads to several pharmacokinetic alterations, including the enlargement of the volume of distribution and/or enhanced clearance for most drugs [26]. The enhanced drug of drugs in cystic fibrosis is generally explained by increased glomerular filtration, increased active tubular secretion, and decreased tubular reabsorption [26,27].

Ganciclovir can be dosed on a milligram-per-kilogram of bodyweight basis as this corresponds well to ganciclovir clearance and the volume of distribution [28]. The other most clearly defined variable affecting the pharmacokinetic parameters is renal function status/creatinine clearance [25,29,30,31].

Based on our results, an LD of 7.988 × height (cm)–992.1 mg followed with a daily MD of 705.4 × eGFR (mL/s)–109.4 mg/day (divided into 2 doses every 12 h) should be optimal. Of course, the administration of an LD only make sense if the LD is higher than a single MD. Therefore, the condition eGFR (mL/s) < 0.023 × height (cm)–2.66 must be met. Thus, LD administration should be considered especially in patients with moderate-to-severe decrease in renal function. The daily MD deducted from the proposed nomogram should be further increased by approximately 50% in patients with cystic fibrosis.

Measured creatinine clearance is not available from most patients at the time when ganciclovir treatment is initiated. Therefore, eGFR according to the CKD-EPI equation was used to individualize ganciclovir MD. The CKD-EPI equation is an up-to-date method for estimating GFR and its superiority in the prediction of MD in other drugs excreted via kidney has been described previously [32,33].

There was no correlation between ganciclovir trough and peak serum levels, nor between hematologic toxicity and nephrotoxicity [20,34]. In our study, ganciclovir exposure (AUC_24_) was associated neither with white blood cell count nor with platelet count. Neither ALT nor GGT were significantly related to the pharmacokinetics of ganciclovir. We also observed no impact of concomitant immunosuppressant treatment or antimycotic therapy on ganciclovir pharmacokinetics. This observation is of interest, since the co-administration of the antifungal voriconazole and ganciclovir was excluded in previous clinical studies [35].

We acknowledge a few limitations of our study. First, we enrolled only one subject with eGFR below 0.5 mL/s; therefore, our dosing recommendation may not be applicable in this subpopulation. Furthermore, the subpopulation of patients with cystic fibrosis is rather limited (n = 5); therefore, the PK and dosing estimates should be considered as pilot data only for this subpopulation.

## 5. Conclusions

Ganciclovir clearance is correlated with creatinine clearance; therefore, ganciclovir should be dosed according to renal function status. Significantly higher bodyweight-normalized CL and lower distribution half-life were observed in patients with cystic fibrosis. As a result, ganciclovir’s daily maintenance dose should be increased by approximately 50% in cystic fibrosis patients. We did not observe any pharmacokinetic drug interactions between ganciclovir and immunosuppressive or antimycotic therapy. Large inter-individual variability of serum levels was observed. This is one of the reasons for supporting TDM. Future studies may aim to identify an appropriate group of patients for ganciclovir dosing according to our nomogram, depending on height and renal function. Our data also provide the basis for the design of a pharmacokinetic model that is needed to more accurately describe the PK/PD relationship in ganciclovir.

## Figures and Tables

**Table 1:** Gradient of mobile phases.

Time (min)	A (%)	B (%)	Flow (mL/min)
0	95	5	0.2
1	0	100	0.4
3	0	100	0.4
3.1	95	5	0.4
3.5	95	5	0.4

**Table 2:** Concentrations measured in the samples at each spiking level in the intra-day and inter-day accuracy and precision study.

Sample	QC 1	QC 2	QC 3	QC 4
	0.1 (ng/mL)	0.5 (ng/mL)	2.5 (ng/mL)	10 (ng/mL)
Intra-Day Accuracy and Precision				
1	0.107	0.47	2.29	9.42
2	0.108	0.47	2.26	9.43
3	0.111	0.47	2.26	9.40
4	0.109	0.47	2.28	9.34
5	0.105	0.47	2.30	9.24
6	0.109	0.47	2.30	9.32
7	0.106	0.48	2.26	9.19
8	0.109	0.47	2.25	9.27
9	0.103	0.47	2.28	9.40
10	0.104	0.46	2.30	9.35
Mean (ng/mL)	0.107	0.47	2.28	9.34
SD (ng/mL)	0.003	0.005	0.021	0.081
CV (%)	2.39	1.16	0.91	0.86
BIAS (%)	7.04	6.21	8.92	6.64
Inter-Day Accuracy and Precision				
1	0.107	0.47	2.23	9.87
2	0.114	0.48	2.45	10.21
3	0.119	0.46	2.26	10.55
4	0.116	0.51	2.79	9.92
5	0.115	0.52	2.36	9.78
6	0.112	0.49	2.48	10.23
7	0.104	0.53	2.74	10.46
8	0.116	0.51	2.77	9.48
9	0.119	0.48	2.69	10.33
10	0.119	0.47	2.61	10.56
Mean (ng/mL)	0.114	0.49	2.54	10.14
SD (ng/mL)	0.005	0.024	0.211	0.36
CV (%)	5.54	4.81	8.32	3.58
BIAS (%)	14.02	1.50	1.50	1.39

**Table 3:** Demographic and laboratory characteristics of the patients (n = 40).

	Median	Interquartile Range	Range
Age (years)	52	46–58	22–71
Body weight (kg)	76	61–86	45–117
Height (cm)	175	168–178	152–197
BSA (m^2^)	1.90	1.73–2.03	1.44–2.51
BMI (kg/m^2^)	25.5	21.7–27.8	15.4–34.9
eGFR creatinine (mL/s/1.73 m^2^)	1.66	1.39–1.84	0.28–2.58
eGFR cystatin C * (mL/s/1.73 m^2^)	0.83	0.56–1.01	0.30–1.88
ALT (μkat/L)	0.56	0.31–1.08	0.15–7.87
GGT (μkat/L)	0.67	0.41–1.17	0.18–15.52
White blood cell count (×10^9^/L)	9.55	7.30–13.25	2.70–23.00
Platelet count (×10^9^/L)	266	187–377	63–530
	**Total count**	**Percentage (%)**	
Sex (M/F)	28/12	70/30	

BSA—body surface area, BMI—body mass index, eGFR—estimated glomerular filtration rate according to the CKD-EPI creatinine or cystatin C equations, ALT—alanine aminotransferase, GGT—gamma-glutamyl transferase. * Analyzed only in a subgroup of 24 patients, in whom the cystatin C level was available.

**Table 4:** Pharmacokinetic analysis of ganciclovir.

PK Parameter	Whole Study Population (n = 40)	CF Patients (n = 5)	Non-CF Patients (n = 35)	*p*-Value CF vs. Non-CF
Vd_c_ (L)	8.96 (7.51–10.30)	6.19 (6.05–7.65)	9.13 (8.36–10.46)	-
Vd_c_ (L/kg)	0.120 (0.110–0.124)	0.129 (0.121–0.130)	0.119 (0.109–0.124)	0.069
Vd (L)	51.7 (32.7–60.1)	39.5 (31.5–59.7)	52.5 (34.3–60.1)	-
Vd (L/kg)	0.65 (0.52–0.73)	0.67 (0.67–1.08)	0.65 (0.50–0.72)	0.1987
CL (L/h)	6.64 (4.27–9.20)	7.39 (6.38–9.29)	6.49 (4.04–9.14)	-
CL (L/h/kg)	0.088 (0.059–0.118)	0.125 (0.112–0.198)	0.081 (0.057–0.106)	0.0127 *
t_1/2_α (h)	0.20 (0.18–0.37)	0.18 (0.16–0.18)	0.20 (0.18–0.44)	0.0318 *
t_1/2_β (h)	4.7 (3.6–5.8)	3.6 (2.8–3.7)	5.2 (3.7–5.9)	0.1448
AUC_24_ (mg × h/L)	104.9 (76.1–154.0)	53.8 (40.9–78.0)	114.2 (85.5–155.9)	-

Data are expressed as median (IQR). Only bodyweight-normalized and independent pharmacokinetic parameters were compared. Statistically significant * CF—cystic fibrosis, Vd_c_—central volume of distribution, Vd—total volume of distribution, CL—clearance, t_1/2_α—distribution half-life, t_1/2_β—elimination half-life, AUC_24_—24 h area under the concentration time curve.

## Data Availability

Data supporting the findings of this study are available from the corresponding author upon reasonable request.

## References

[1] Hubacek P., Virgili A., Ward K.N., Pohlreich D., Keslova P., Goldova B., Markova M., Zajac M., Cinek O., Nacheva E.P. (2009). HHV-6 DNA throughout the tissues of two stem cell transplant patients with chromosomally integrated HHV-6 and fatal CMV pneumonitis. Br. J. Haematol..

[2] Kurihara C., Fernandez R., Safaeinili N., Akbarpour M., Wu Q., Budinger G.R.S., Bharat A. (2019). Long-Term Impact of Cytomegalovirus Serologic Status on Lung Transplantation in the United States. Ann. Thorac. Surg..

[3] Kotton C.N., Kumar D., Caliendo A.M., Huprikar S., Chou S., Danziger-Isakov L., Humar A., The Transplantation Society International CMV Consensus Group (2018). The Third International Consensus Guidelines on the Management of Cytomegalovirus in Solid-organ Transplantation. Transplantation.

[4] Ho S.A., Slavin M., Roberts J.A., Yong M. (2021). Optimization of Ganciclovir use in allogeneic hematopoietic cell transplant recipients—The role of therapeutic drug monitoring. Expert Rev. Anti-Infect. Ther..

[5] Perrottet N., Decosterd L.A., Meylan P., Pascual M., Biollaz J., Buclin T. (2009). Valganciclovir in adult solid organ transplant recipients: Pharmacokinetic and pharmacodynamic characteristics and clinical interpretation of plasma concentration measurements. Clin. Pharmacokinet..

[6] Gilbert C., Boivin G. (2005). Human cytomegalovirus resistance to antiviral drugs. Antimicrob. Agents Chemother..

[7] Razonable R.R. (2013). Management strategies for cytomegalovirus infection and disease in solid organ transplant recipients. Infect. Dis. Clin. N. Am..

[8] Davis C.L., Springmeyer S., Gmerek B.J. (1990). Central nervous system side effects of ganciclovir. N. Engl. J. Med..

[9] Chambers D.C., Zuckermann A., Cherikh W.S., Harhay M.O., Hayes D., Hsich E., Khush K.K., Potena L., Sadavarte A., Singh T.P. (2020). The International Thoracic Organ Transplant Registry of the International Society for Heart and Lung Transplantation: 37th adult lung transplantation report—2020; focus on deceased donor characteristics. J. Heart Lung Transplant..

[10] Snell G.I., Kotsimbos T.C., Levvey B.J., Skiba M., Rutherford D.M., Kong D.C., Williams T.J., Krum H. (2000). Pharmacokinetic assessment of oral ganciclovir in lung transplant recipients with cystic fibrosis. J. Antimicrob. Chemother..

[11] Crumpacker C.S. (1996). Ganciclovir. N. Engl. J. Med..

[12] Faulds D., Heel R.C. (1990). Ganciclovir: A review of its antiviral activity, pharmacokinetic properties and therapeutic efficacy in cytomegalovirus infections. Drugs.

[13] Du Bois D., Du Bois E.F. (1989). A formula to estimate the approximate surface area if height and weight be known. Nutrition.

[14] Inker L.A., Schmid C.H., Tighiouart H., Eckfeldt J.H., Feldman H.I., Greene T., Kusek J.W., Manzi J., Van Lente F., Zhang Y.L. (2012). Estimating glomerular filtration rate from serum creatinine and cystatin C. N. Engl. J. Med..

[15] Garrow J.S. (1986). Quetelet index as indicator of obesity. Lancet.

[16] U.S. Department of Health and Human Services, Food and Drug Administration (2018). Guidance for Industry: Bioanalytical Method Validation. U.S. https://www.fda.gov/files/drugs/published/Bioanalytical-Method-Validation-Guidance-for-Industry.pdf.

[17] Sommadossi J.P., Bevan R., Ling T., Lee F., Mastre B., Chaplin M.D., Nerenberg C., Koretz S., Buhles W.C. (1988). Clinical pharmacokinetics of ganciclovir in patients with normal and impaired renal function. Rev. Infect. Dis..

[18] Bonett D.G., Price R.M. (2002). Statistical inference for a linear function of medians: Confidence intervals, hypothesis testing, and sample size requirements. Psychol. Methods.

[19] Sima M., Pokorna P., Hronova K., Slanar O. (2015). Effect of co-medication on the pharmacokinetic parameters of phenobarbital in asphyxiated newborns. Physiol. Res..

[20] Ritchie B.M., Barreto J.N., Barreto E.F., Crow S.A., Dierkhising R.A., Jannetto P.J., Tosh P.K., Razonable R.R. (2019). Relationship of Ganciclovir Therapeutic Drug Monitoring with Clinical Efficacy and Patient Safety. Antimicrob. Agents Chemother..

[21] Trachuk P., Bartash R., Abbasi M., Keene A. (2020). Infectious Complications in Lung Transplant Recipients. Lung.

[22] Lurain N.S., Chou S. (2010). Antiviral drug resistance of human cytomegalovirus. Clin. Microbiol. Rev..

[23] Stockmann C., Roberts J.K., Knackstedt E.D., Spigarelli M.G., Sherwin C.M. (2015). Clinical pharmacokinetics and pharmacodynamics of ganciclovir and valganciclovir in children with cytomegalovirus infection. Expert Opin. Drug Metab. Toxicol..

[24] Galar A., Valerio M., Catalan P., Garcia-Gonzalez X., Burillo A., Fernandez-Cruz A., Zatarain E., Sousa-Casasnovas I., Anaya F., Rodriguez-Ferrero M.L. (2021). Valganciclovir-Ganciclovir Use and Systematic Therapeutic Drug Monitoring. An Invitation to Antiviral Stewardship. Antibiotics.

[25] Martson A.G., Edwina A.E., Burgerhof J.G.M., Berger S.P., de Joode A., Damman K., Verschuuren E.A.M., Blokzijl H., Bakker M., Span L.F. (2021). Ganciclovir therapeutic drug monitoring in transplant recipients. J. Antimicrob. Chemother..

[26] De Sutter P.J., Gasthuys E., Van Braeckel E., Schelstraete P., Van Biervliet S., Van Bocxlaer J., Vermeulen A. (2020). Pharmacokinetics in Patients with Cystic Fibrosis: A Systematic Review of Data Published Between 1999 and 2019. Clin. Pharmacokinet..

[27] Jouret F., Bernard A., Hermans C., Dom G., Terryn S., Leal T., Lebecque P., Cassiman J.J., Scholte B.J., de Jonge H.R. (2007). Cystic fibrosis is associated with a defect in apical receptor-mediated endocytosis in mouse and human kidney. J. Am. Soc. Nephrol..

[28] Scott J.C., Partovi N., Ensom M.H. (2004). Ganciclovir in solid organ transplant recipients: Is there a role for clinical pharmacokinetic monitoring?. Ther. Drug Monit..

[29] Lefeuvre S., Chevalier P., Charpentier C., Zekkour R., Havard L., Benammar M., Amrein C., Boussaud V., Lillo-Le Louet A., Guillemain R. (2010). Valganciclovir prophylaxis for cytomegalovirus infection in thoracic transplant patients: Retrospective study of efficacy, safety, and drug exposure. Transpl. Infect. Dis..

[30] Murray B.M. (1997). Management of cytomegalovirus infection in solid-organ transplant recipients. Immunol. Investig..

[31] Palacio-Lacambra M.E., Comas-Reixach I., Blanco-Grau A., Sune-Negre J.M., Segarra-Medrano A., Montoro-Ronsano J.B. (2018). Comparison of the Cockcroft-Gault, MDRD and CKD-EPI equations for estimating ganciclovir clearance. Br. J. Clin. Pharmacol..

[32] Sima M., Hartinger J., Cikankova T., Slanar O. (2018). Estimation of once-daily amikacin dose in critically ill adults. J. Chemother..

[33] Sima M., Hartinger J., Stenglova Netikova I., Slanar O. (2018). Creatinine Clearance Estimations for Vancomycin Maintenance Dose Adjustments. Am. J. Ther..

[34] Wiltshire H., Paya C.V., Pescovitz M.D., Humar A., Dominguez E., Washburn K., Blumberg E., Alexander B., Freeman R., Heaton N. (2005). Pharmacodynamics of oral ganciclovir and valganciclovir in solid organ transplant recipients. Transplantation.

[35] Rousseau A., Monchaud C., Debord J., Vervier I., Estenne M., Thiry P., Marquet P. (2003). Bayesian forecasting of oral cyclosporin pharmacokinetics in stable lung transplant recipients with and without cystic fibrosis. Ther. Drug Monit..

